# Deep learning accurately and reliably segments pelvic vascular structure in CT scans of gynecologic cancer patients

**DOI:** 10.3389/fonc.2026.1687859

**Published:** 2026-04-16

**Authors:** Hyeonseung Kim, Min Jin Jeong, Kyo Yeong Koo, Youn Jin Choi, Eun Seo Heo, Woohyun Nam, Sangyun Kang, U-Young Lee, Yi-Suk Kim, Keun Ho Lee, Chan-Ung Park

**Affiliations:** 1 SKIA, Inc., Seoul, Republic of Korea; 2 Department of Obstetrics and Gynecology, Eunpyeong St. Mary’s Hospital, College of Medicine, The Catholic University of Korea, Seoul, Republic of Korea; 3 Department of Obstetrics and Gynecology, Seoul St. Mary’s Hospital, The Catholic University of Korea, Seoul, Republic of Korea; 4 The Catholic Institute for Applied Anatomy, Department of Anatomy, College of Medicine, The Catholic University of Korea, Seoul, Republic of Korea

**Keywords:** blood vessels, computed tomography angiography, deep learning, female, genital neoplasms, iliac artery

## Abstract

**Introduction:**

Accurate identification of pelvic vascular structures is critical for safe and effective gynecologic cancer surgery, yet intraoperative recognition of the internal iliac artery, its branches, and corresponding venous pathways remains challenging due to complex anatomy and inter-individual variability. This study investigates the feasibility of fully automated, multi-class segmentation of both major pelvic vessels and smaller branches of the internal iliac artery and internal iliac vein using the nnU-Net v2 deep learning framework.

**Methods:**

Contrast-enhanced computed tomography angiography datasets from 47 patients were used for model training and evaluation. The nnU-Net v2 deep learning framework was employed to perform fully automated segmentation across nine vascular categories. These targets included major vessels, the internal iliac artery and vein, and an "other vessels" category consolidating surgically critical smaller branches, such as the obturator, uterine, and gluteal vessels. Model performance was quantitatively evaluated against ground truth annotations using the Dice Similarity Coefficient and Mean Surface Distance.

**Results:**

The model achieved high segmentation accuracy for large-diameter vessels such as the aorta and iliac arteries, with average Dice similarity coefficients exceeding 0.83 for arterial structures and 0.75 for venous structures. Smaller branches exhibited lower overlap scores but maintained anatomically coherent and clinically recognizable patterns, particularly for the obturator and gluteal arteries.

**Discussion:**

These findings demonstrate the potential of automated 3D vascular segmentation to enhance preoperative planning, anatomical education, and intraoperative safety in complex pelvic procedures. Furthermore, this represents the first systematic evaluation of the segmentation of major branches of the internal iliac artery in gynecologic oncology patients.

## 1 Introduction

Gynecologic cancers, including cervical, ovarian, and endometrial malignancies, constitute a significant global health challenge, with an estimated annual incidence of over 1.3 million new cases and 600,000 deaths worldwide (GLOBOCAN 2024) (1). Despite advances in screening and systemic therapies, surgery remains a cornerstone of both diagnosis and treatment, playing a critical role in staging, cytoreduction, and local disease control (2). In this context, accurate intraoperative identification of anatomical structures is essential to avoid complications such as hemorrhage, vascular or ureteral injury, and incomplete lymphadenectomy.

Among various anatomical landmarks, the internal iliac artery, the internal iliac vein, and their respective branches are of critical importance in gynecologic oncology surgery. During procedures such as radical hysterectomy and pelvic lymphadenectomy, these vessels not only supply major pelvic organs, including the uterus, bladder, rectum, and vagina, but also delineate critical dissection planes (3). Moreover, the internal iliac artery serves as a key anatomical reference for accessing the obturator fossa and pararectal/paravesical lymph node regions. However, the pelvic vasculature displays considerable inter-individual variability, including aberrant branching patterns and atypical vessel origins, which can increase the risk of neurovascular and ureteral injury during surgery (4). If the detailed vascular branching patterns of the internal iliac artery and its subdivisions can be reliably visualized during preoperative planning, it may contribute to reducing intraoperative blood loss and improving surgical safety.

Accordingly, the increasing integration of preoperative imaging, such as contrast-enhanced computed tomography (CT) (5, 6) and pelvic magnetic resonance imaging (MRI) (7, 8), into clinical workflows has highlighted the need for automated, high-precision segmentation tools to enhance anatomical understanding and support individualized surgical planning (9). Nevertheless, current workflows largely rely on manual interpretation or static 2D visualization, which are insufficient for capturing the complex three-dimensional relationships between pelvic vessels and adjacent organs.

Although deep learning has significantly advanced medical image analysis, most existing segmentation tools remain semi-automatic, organ-specific, or require extensive parameter tuning, limiting their reproducibility and generalizability across imaging modalities and institutions (10–12). These limitations present a significant barrier to the routine application of AI-based segmentation in complex oncologic surgeries such as those for gynecologic cancers.

In recent years, nnU-Net has emerged as a fully automated segmentation tool that consistently achieves top performance on diverse medical imaging challenges without requiring any manual parameter tuning (13). This data-driven design improves usability by eliminating the need for manual parameter tuning. The release of nnU-Net v2 has further enhanced its scalability (14). Despite its strong performance and flexibility, its practical application has remained mostly confined to single-organ segmentation or disease-specific contexts.

While some prior studies have applied deep learning to segment cervical or ovarian tumors (15–17), few have addressed vascular structures essential for surgical planning. Most existing vascular segmentation efforts have focused on the aorta or the common and external iliac arteries (18, 19). Prior research has explored pelvic vascular segmentation; however, systematic evaluation of the segmentation accuracy of the internal iliac artery and its branches using clinical imaging data from gynecologic cancer patients remains limited.

In this study, we adopted the publicly available nnU-Net v2 deep learning framework to perform multi-class segmentation of both arterial and venous pelvic vessels, including the aorta (Ao), common iliac artery (CIA), external iliac artery (EIA), internal iliac artery (IIA), inferior vena cava (IVC), common iliac vein (CIV), external iliac vein (EIV), internal iliac vein (IIV), as well as other vascular structures.

To address this gap, this study investigates the clinical feasibility of automated 3D segmentation for a surgically critical yet underexplored anatomical target by applying a state-of-the-art deep learning framework to preoperative CT images of gynecologic oncology patients. The major contributions of this work are as follows:

- This is the first study to systematically assess the feasibility and accuracy of automated segmentation specifically targeting the IIA and its anatomically complex branches using clinical data from gynecologic oncology patients.
- The publicly available nnU-Net v2 deep learning framework was adopted to perform fully automated, multi-class segmentation of both arterial and venous pelvic vessels, including the aorta (Ao), common iliac artery (CIA), external iliac artery (EIA), internal iliac artery (IIA), inferior vena cava (IVC), common iliac vein (CIV), external iliac vein (EIV), internal iliac vein (IIV), and other smaller vascular structures.
- By successfully segmenting this surgically critical vascular region, the proposed approach provides a foundation for improving preoperative understanding, anatomical education, and intraoperative safety in complex gynecologic cancer surgeries.

Following the introduction of our primary contributions, we explore related works to provide a comprehensive background for our study. We then describe the proposed methodology, outlining the data collection process and the training procedures for the segmentation model. The quantitative and qualitative results of this approach are subsequently presented and analyzed. Finally, we discuss the clinical implications of our findings before concluding the paper.

## 2 Related works

### 2.1 Deep learning architectures for medical image segmentation

Deep learning architectures for medical image segmentation have been widely adopted to address the unique challenges of extracting thin, continuous, and highly branched 3D vascular structures. The foundational 3D U-Net (20) revolutionized 3D medical image analysis through its symmetric encoder-decoder structure and 3D convolutional kernels. When applied to vascular segmentation, 3D U-Net is highly effective at extracting local high-contrast features from large vessels, but its inherently limited receptive field often struggles to maintain the structural continuity of tortuous or distal branch vessels (21).

To overcome these local structural disconnections and capture long-range spatial dependencies, research has shifted toward integrating attention mechanisms and Vision Transformers (ViTs) into medical image analysis. A prominent milestone in this broader transition is Swin UNETR (22), which employs a shifted window (Swin) Transformer encoder connected to a CNN-based decoder. By computing self-attention across multi-scale patches, Swin UNETR effectively models the global anatomical context. This capability makes it highly advantageous when applied to tracing complex, long-range vascular networks (23). Building upon this trend, Imran et al. (24) introduced CIS-UNet. By applying Context-aware Shifted Window Self-Attention, CIS-UNet successfully distinguished closely related vascular boundaries, performing precise multi-class segmentation of the aorta and 13 major branches (a total of 14 classes), including the common, external, and internal iliac arteries.

The evolution of medical image segmentation also encompasses fully automated and self-configuring frameworks that establish robust, state-of-the-art baselines across diverse anatomies. The nnU-Net v2 framework (14) systematically optimizes the fundamental 3D U-Net topology, dynamically adapting preprocessing, network architectures, and hyperparameters to the specific dataset. This data-driven optimization consistently captures delicate structural details with high accuracy across various organs and tissues, eliminating the need for manual tuning.

### 2.2 Pelvic vasculature in gynecologic oncology

Despite the impressive performance of models like CIS-UNet in segmenting major aortic branches, comprehensive studies targeting the intricate pelvic vascular network remain remarkably sparse. While CIS-UNet included the internal iliac artery (IIA) in its 14 target classes, its scope ended there. It did not address the complex, surgically critical subdivisions of the IIA and internal iliac vein (IIV)—such as the obturator, uterine, and gluteal vessels—which are highly variable and challenging to identify intraoperatively.

Existing deep learning applications in gynecologic oncology have predominantly focused on tumor segmentation rather than vascular structures, leaving fully automated 3D segmentation of the variable and fine-detailed branches of the IIA and IIV largely unaddressed (16, 17). This represents a research gap, as no systematic deep learning framework has yet been validated for this anatomically complex and surgically critical pelvic vascular territory in gynecologic oncology patients.

To bridge this gap, our study fine-tunes the nnU-Net v2 framework specifically on a gynecologic oncology CTA dataset. This domain-specific fine-tuning overcomes the extreme anatomical variability of the pelvic network, enabling fully automated, multi-class 3D segmentation of both major pelvic vessels and the previously neglected intricate branches of the IIA and IIV for safe surgical planning.

## 3 Materials and methods

### 3.1 Dataset collection

The dataset used in this study consisted of contrast-enhanced CT angiography (CTA) scans collected from patients who visited the Department of Obstetrics and Gynecology at Seoul St. Mary’s Hospital between 2006 and 2023. Eligible patients were those diagnosed with gynecologic cancers (ICD-10 codes C539, C541, C560, C561, C569), benign tumors (ICD-10 codes D259, D279), or nonspecific abdominal/pelvic pain (ICD-10 codes R100, R1030, R1031, R1039, R1049). The study was approved by the Institutional Review Board (IRB No. 20230427-R-121), and due to its retrospective nature, no informed consent from patients was required.

**Figure 1:**
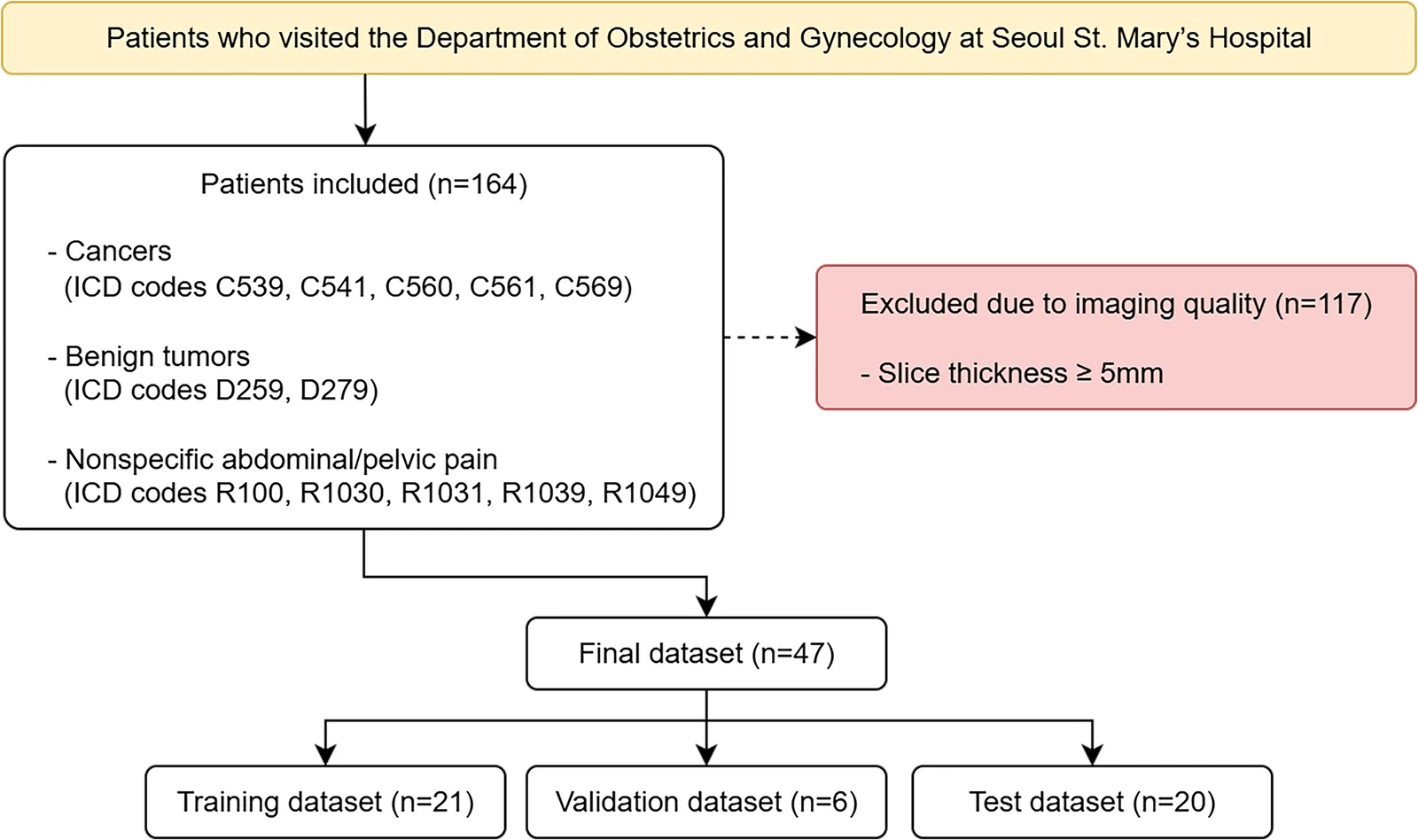
Flowchart illustrating the inclusion process of CTA datasets used for deep learning model training and evaluation.

From a total of 164 CTA scans, 32 scans acquired with a slice thickness of 2 mm and 15 scans with submillimeter (≤1 mm) slice thickness were selected for model training and evaluation. The training was conducted using the 2 mm CTA scans. However, the number of test samples was initially insufficient to support reliable performance evaluation. To address this, all 15 scans from the submillimeter group were resampled to a 2 mm slice thickness using standard volumetric interpolation techniques (**Figure 1**). The uniformity in equipment and post-processing resolution minimized domain shift and allowed for more robust model evaluation. This dataset preparation strategy reflects a realistic clinical imaging environment while ensuring adequate training and validation performance for deep learning–based analysis. All CTA images were acquired using the SOMATOM Force CT scanner (Siemens Healthineers, Germany).

### 3.2 Ground truth annotation

**Figure 2:**
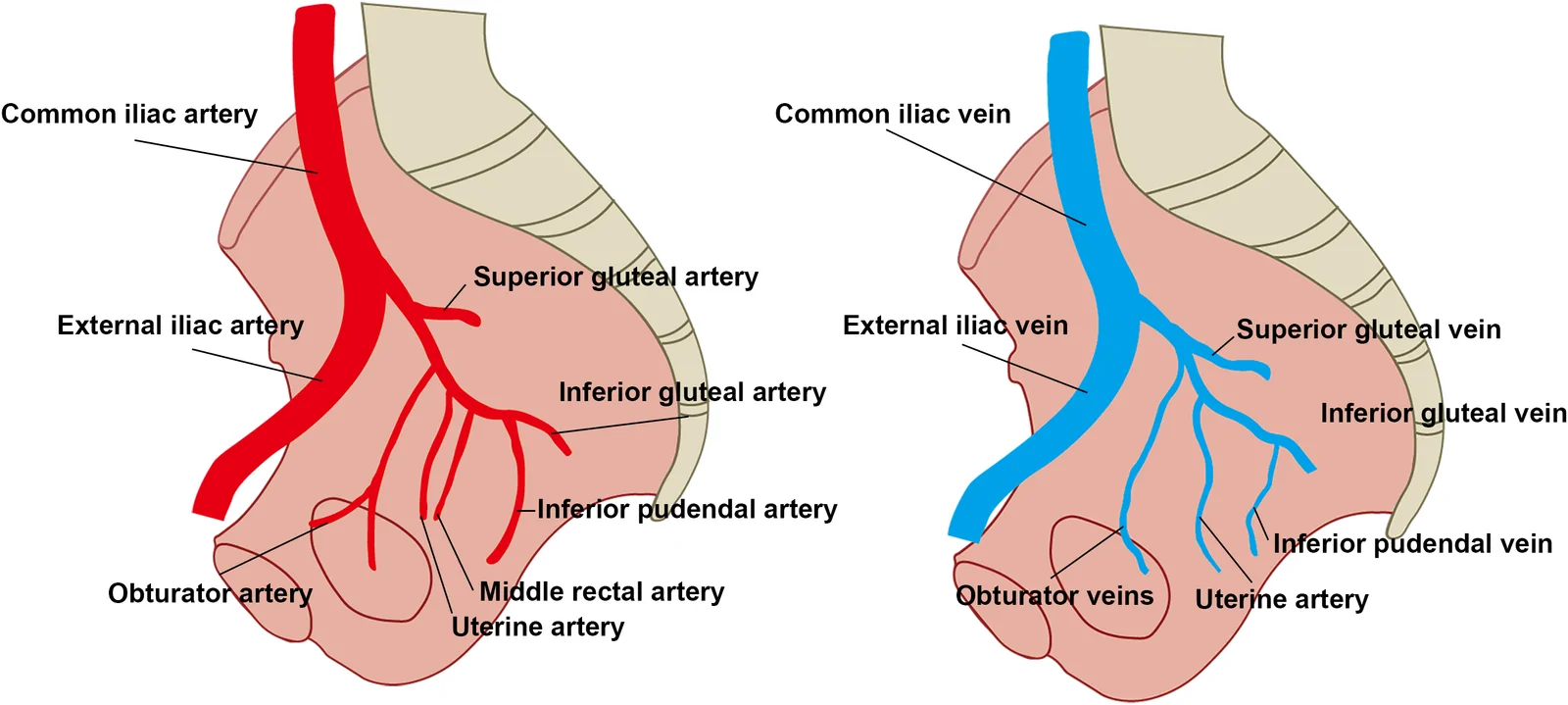
Vascular structures included in the other vessels category. (Left) Major branches of the IIA included in the other vessels, along with the CIA and EIA. (Right) Major sources of the IIV included in the other vessels, along with the CIV and EIV.

Accurate automated segmentation of major vessels significantly impacts surgical planning. This study defines the primary vascular structures for gynecologic cancer surgery as the Ao, CIA, EIA, IIA, IVC, CIV, EIV, IIV, and a consolidated “other vessels” category. This latter group encompasses major branches of the IIA and IIV, specifically the uterine, middle rectal, obturator, and gluteal arteries and veins (**Figure 2**).

Identifying these specific branches is critical during pelvic dissection and vascular control. Including them as segmentation targets directly enhances the clinical applicability of preoperative planning and intraoperative navigation in gynecologic oncology.

Ground truth (GT) annotations reflecting this vascular classification were manually generated using 3D Slicer software (25) by three medical imaging specialists with over three years of experience. A clinician subsequently reviewed five randomly selected cases to validate annotation quality. Within the “other vessels” category, regions with intertwined arterial and venous paths often precluded distinct separation. Both vessel types in these highly complex areas were merged under the unified “other vessels” label.

### 3.3 Model architecture and training

**Figure 3:**
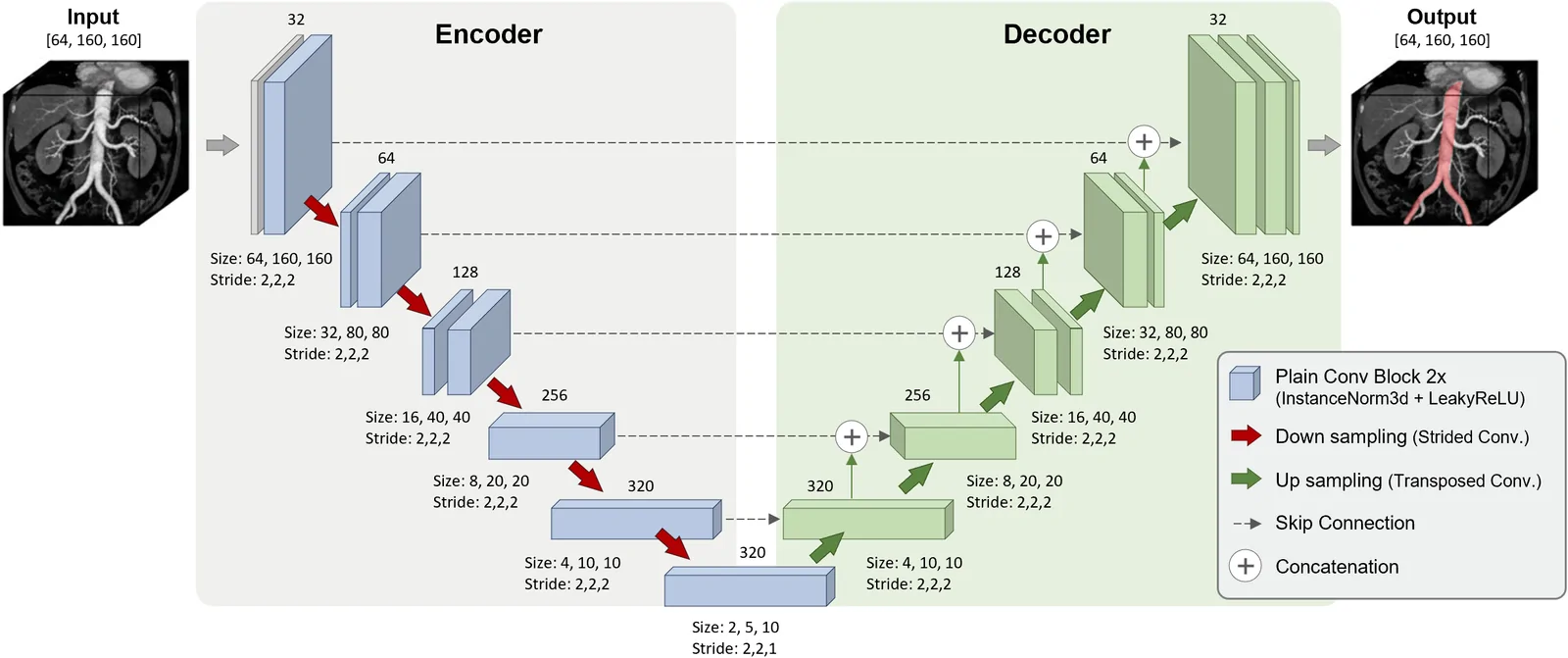
Architecture of nnU-Net v2 for multi-class pelvic vascular segmentation. The model adopts a six–stage 3D encoder–decoder structure based on the PlainConvUNet design.

As illustrated in **Figure 1**, a total of 47 IRB-approved CTA datasets were randomly partitioned into 21 cases for training, 6 for validation, and 20 for independent performance evaluation. The network follows a 3D U-Net-like architecture with six encoding stages, progressively increasing the number of feature channels from 32 to 320. Convolutions use a 3 × 3 × 3 kernel, with stride 2 applied for downsampling in the spatial dimensions, while the deepest stage employs a stride of 1 along the axial dimension to preserve resolution. Instance normalization and LeakyReLU activation are incorporated in all layers (**Figure 3**). The model was trained using stochastic gradient descent (SGD) with an initial learning rate of 0.01 and a polynomial learning rate scheduler, optimizing a combined Dice and cross-entropy loss over 2000 epochs with early stopping to prevent overfitting. Hyperparameters were set as follows: patch size 160 × 160 × 64, voxel spacing 0.77 × 0.77 × 2 mm, and batch size 2. During the training phase, data augmentation techniques, including translation, rotation, and cropping, were applied to improve model robustness. All experiments were conducted on a workstation equipped with an AMD Ryzen 7800X3D CPU (8 cores), 64 GB RAM, and a single NVIDIA GeForce RTX 4090 (24 GB VRAM). The mean inference time was 1.11 seconds per patch with a batch size of 1.

To rigorously evaluate the proposed approach, three established architectures—3D U-Net, Swin UNETR, and CIS-UNet—were selected as baseline models and identically fine-tuned on the same gynecologic oncology CTA dataset. The detailed comparative results of these fine-tuned models are presented in Section 4.1, Quantitative Evaluation.

## 4 Results

### 4.1 Quantitative evaluation

The automated segmentation performance was quantitatively measured on 20 test cases using Dice Similarity Coefficient (DSC) and Mean Surface Distance (MSD, in millimeters). DSC is widely used to evaluate volumetric overlap between the automated segmentation mask and GT, while MSD allows for the quantification of distance-based boundary errors, offering complementary insights into segmentation quality.


$$DSC(A,\;B)=\frac{2\;|A\cap B|}{\left|A|+|B\right|}$$

$$MSD=\frac{1}{N_{SA}+N_{SB}}\{\sum_{a\in SA}\underset{b\in SB}{\min}(dist(a,\;b))\;+\;\sum_{b\in SB}\underset{a\in SA}{\min}(dist(a,\;b))\}$$

In the DSC and MSD formula, A and B represent the sets of voxels in the ground truth (GT) and automated segmentation, respectively. A DSC value of 1.0 indicates perfect overlap, whereas 0 indicates no overlap of voxels. In the MSD calculation, SA and SB represent the sets of points lying on the surfaces of the GT and automated segmentation volumes, respectively. $N_{SA}$ and $N_{SB}$ denote the total number of surface points in SA and SB. The MSD is computed as the average of the shortest Euclidean distances from each point on SA to the nearest point on SB and vice versa. This bidirectional measurement captures boundary alignment errors in millimeters, with lower values indicating closer agreement between the predicted and reference surfaces.

**Figure 4:**
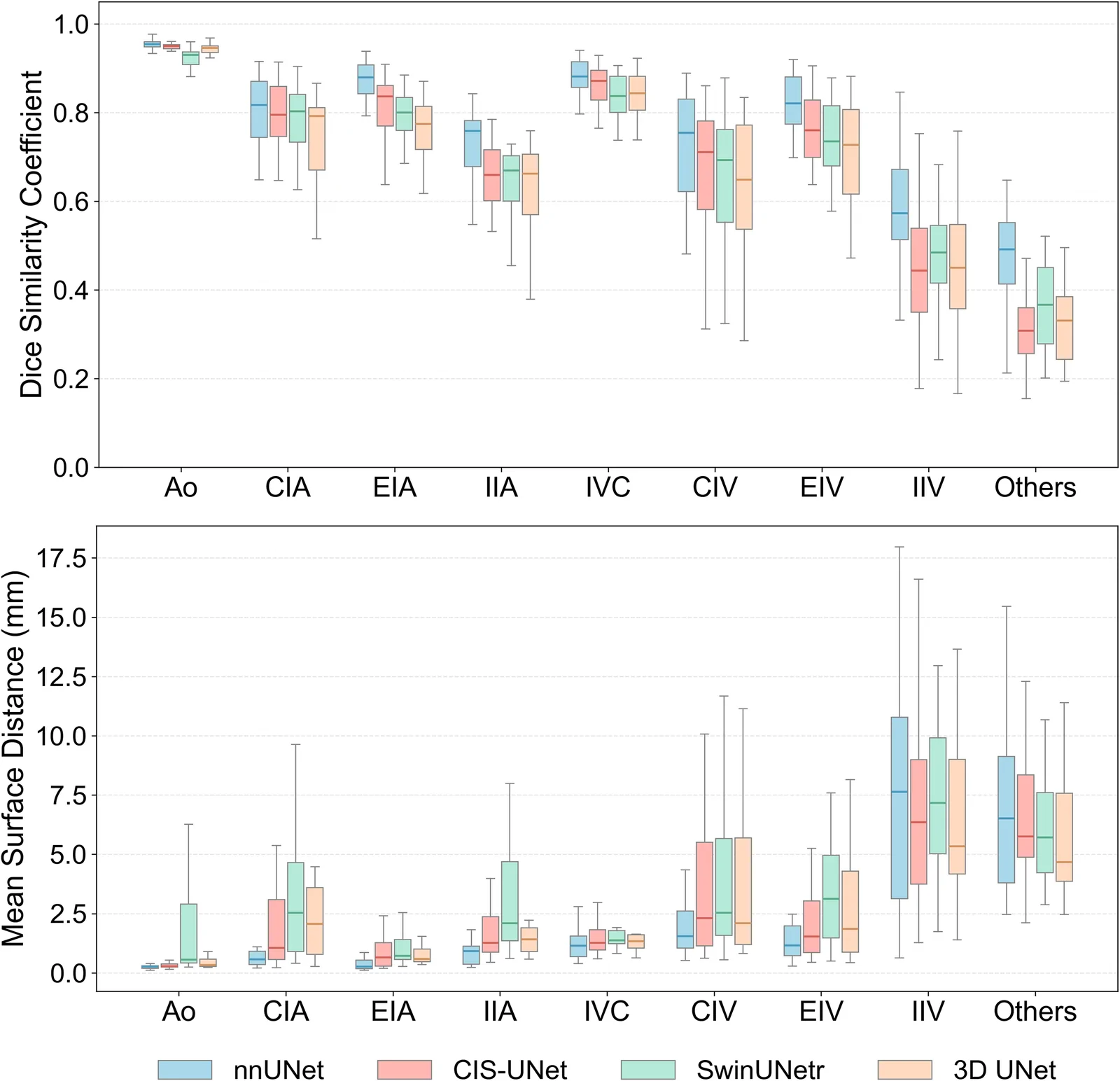
Distribution of segmentation performance across all evaluated models. The box plots illustrate the distribution of (Top) Dice Similarity Coefficients and (Bottom) Mean Surface Distances for the nine target vascular classes. The proposed nnU-Net v2 demonstrates the most compact interquartile ranges and the highest median scores, indicating superior accuracy and robustness compared to the baseline models.

**Tables 1** and **2** summarize the quantitative comparison of the automated segmentation masks against the ground truth (GT) across the four evaluated models. The nnU-Net v2 framework achieved the highest overall performance, yielding an average Dice Similarity Coefficient (DSC) of 0.756 and an average Mean Surface Distance (MSD) of 2.847 mm across all nine target classes. The baseline architectures—3D U-Net, Swin UNETR, and CIS-UNet—recorded average DSCs of 0.664, 0.681, and 0.689, respectively, indicating the effectiveness of nnU-Net v2 in adapting to complex pelvic anatomy. As illustrated in **Figure 4**, nnU-Net v2 consistently achieved the highest median DSC across all vessel classes and the lowest median MSD in major vessels (e.g., Ao, CIA, EIA, IVC, CIV, and EIV), demonstrating superior accuracy and robustness compared to the baseline models. However, all evaluated models exhibited a broader performance variance and elevated boundary errors in the other vessels category, highlighting the inherent predictive challenges associated with these highly intricate and small-diameter branches.

**Table 1:** Dice similarity coefficient comparison of vessel segmentation models.

Vessels	Model performance (DSC)			
	3D U-Net	Swin UNETR	CIS-UNet	nnU-Net v2
Aorta	0.941	0.921	0.945	**0.952**
Common iliac artery	0.731	0.744	0.759	**0.788**
External iliac artery	0.761	0.782	0.815	**0.867**
Internal iliac artery	0.605	0.615	0.635	**0.720**
Inferior vena cava	0.833	0.834	0.859	**0.876**
Common iliac vein	0.622	0.659	0.664	**0.722**
External iliac vein	0.709	0.736	0.754	**0.823**
Internal iliac vein	0.455	0.483	0.456	**0.585**
Other vessels	0.323	0.360	0.316	**0.467**
Average	0.664	0.681	0.689	**0.756**

**Table 2:** Mean surface distance comparison of vessel segmentation models.

Vessels	Model performance (MSD, mm)			
	3D U-Net	Swin UNETR	CIS-UNet	nnU-Net v2
Aorta	0.602	2.231	0.470	**0.347**
Common iliac artery	2.546	3.729	2.267	**0.991**
External iliac artery	0.887	1.056	**0.835**	1.219
Internal iliac artery	1.814	3.475	2.030	**1.082**
Inferior vena cava	1.494	1.580	1.487	**1.393**
Common iliac vein	4.576	4.178	4.139	**3.625**
External iliac vein	2.790	3.723	2.392	**1.694**
Internal iliac vein	8.304	8.179	**7.140**	8.024
Other vessels	**5.789**	6.036	6.292	7.248
Average	3.200	3.799	3.006	**2.847**

The aorta (Ao) was segmented with high precision across most models, with nnU-Net v2 reaching a DSC of 0.952 and an MSD of 0.347 mm. Among the major iliac arteries (CIA, EIA) and veins (IVC, CIV, EIV), nnU-Net v2 consistently demonstrated improved volumetric overlap compared to the baseline models, maintaining DSCs above 0.72. While CIS-UNet yielded a slightly lower MSD for the external iliac artery (0.835 mm vs. 1.219 mm), nnU-Net v2 provided higher volumetric agreement with a DSC of 0.867 compared to 0.815 for the same structure.

Performance differences became most distinct in the surgically relevant smaller branches. The baseline models struggled with the internal iliac artery (IIA), recording DSCs between 0.605 and 0.635, whereas nnU-Net v2 reached 0.720 alongside an MSD of 1.082 mm. This advantage in volumetric agreement extended to the internal iliac vein (IIV) and the highly complex “other vessels” category, where nnU-Net v2 consistently achieved the highest DSCs among all evaluated models. The inherent tortuosity and small diameter of these minute vessels naturally present a challenge for boundary estimation, leading to elevated MSD values across all models, yet nnU-Net v2 consistently preserved the strongest overall structural overlap.

### 4.2 Qualitative evaluation

**Figure 5:**
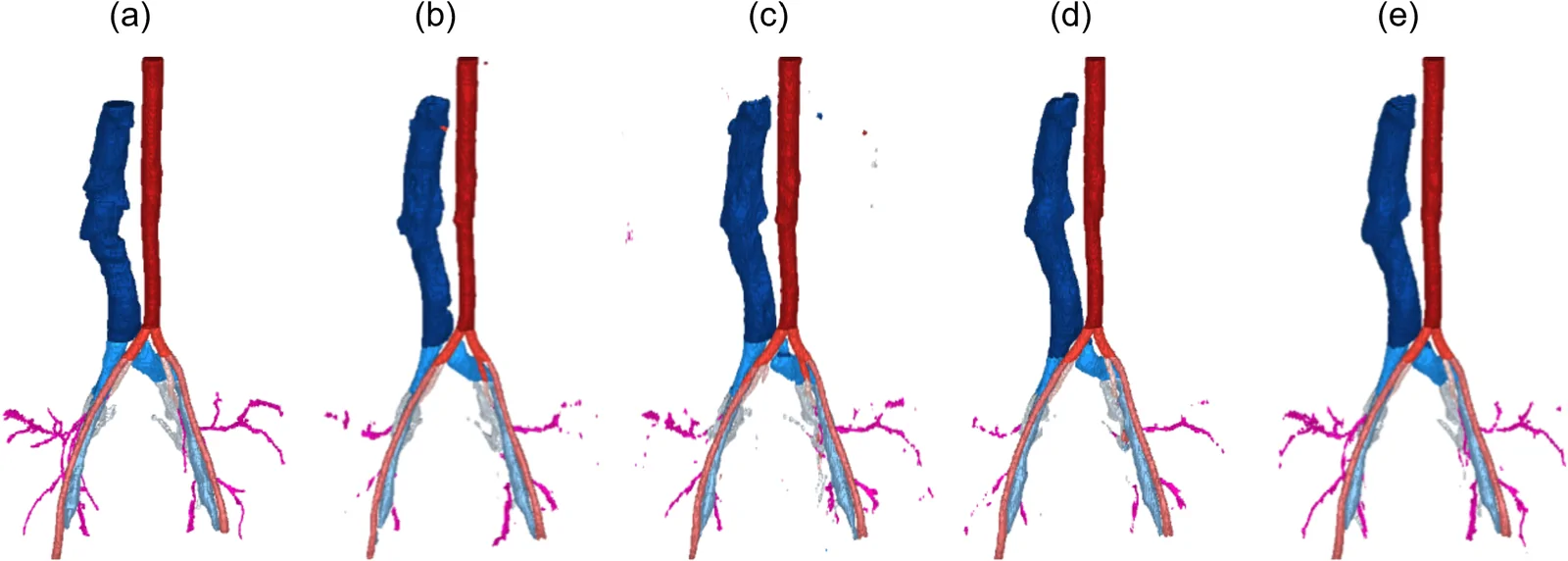
Qualitative comparison of 3D vascular segmentation across evaluated models. **(A)** Ground truth, followed by automated segmentation results produced by **(B)** 3D U-Net, **(C)** Swin UNETR, **(D)** CIS-UNet, and **(E)** the proposed nnU-Net v2. Arterial structures are shown in shades of red, venous structures in shades of blue, and other vessels in purple.

To further assess the anatomical validity of the segmentation results, both GT and automated segmentation masks were reconstructed in 3D and visualized together with the CT-derived bony structures (**Figure 5**).

In the majority of cases, automated segmentations closely followed the expected vascular architecture, demonstrating continuous connections from the Ao to the CIA, from the CIA to the EIA and IIA, and from the IIA to its peripheral branches categorized as other vessels. Similarly, venous pathways were well represented, showing convergence from the other vessels to the IIV, and subsequently through the EIV and CIV to the IVC.

**Figure 6:**
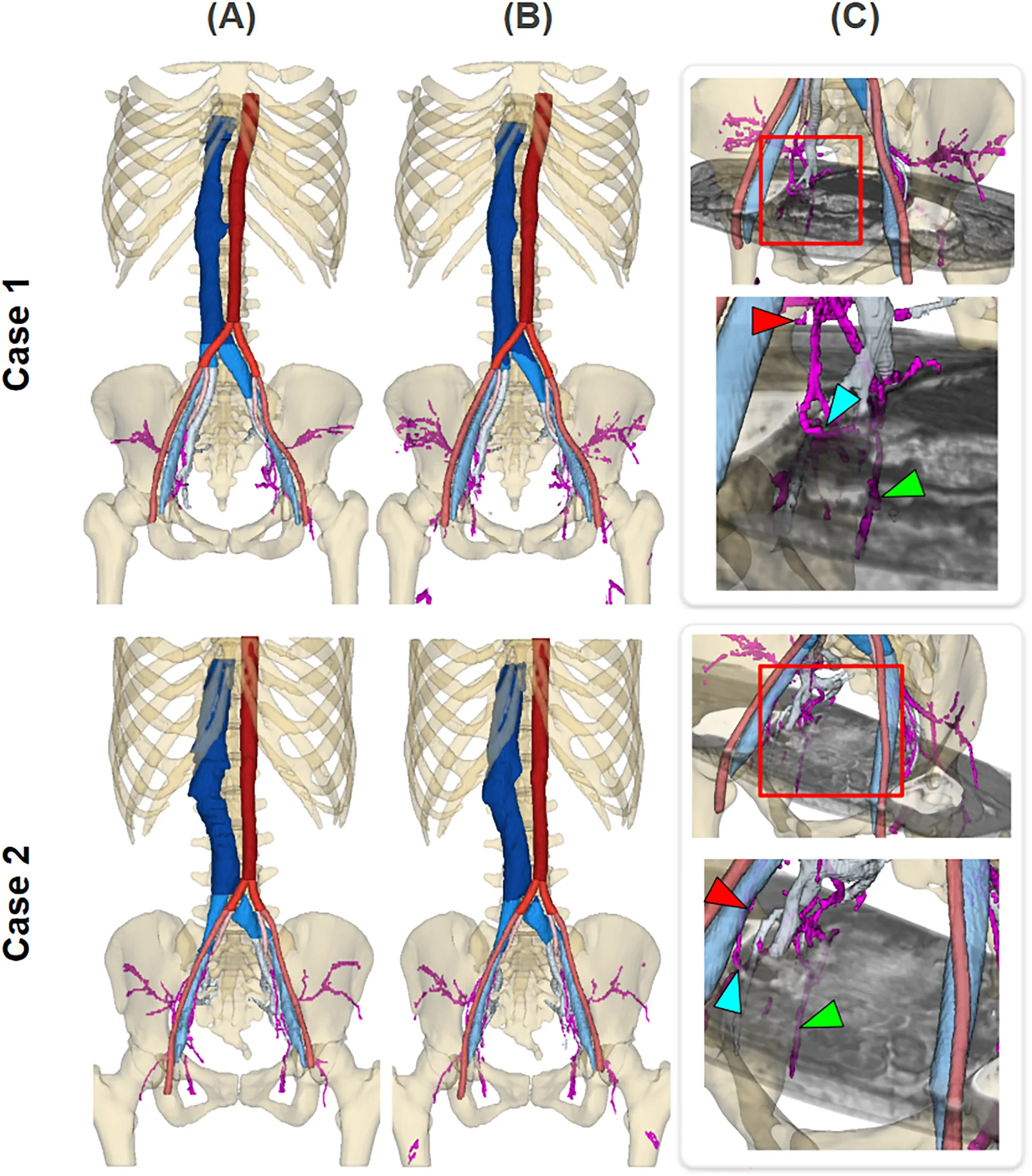
Qualitative comparison of automated segmentation results. 3D visualization of the **(A)** Ground truth and **(B)** the proposed nnU-Net segmentation mask. Arterial structures are shown in shades of red, venous structures in shades of blue, and other vessels in purple. **(C)** Enlarged view of the other vessels category in automated segmentation mask. The red, cyan, and green pin indicates the obturator artery, the uterine artery, and the internal pudendal artery, respectively.

Despite the relatively low DSC (0.467) for the other vessels category, which includes major branches with smaller vessel diameters derived from the IIA and IIV, qualitative inspection revealed anatomically coherent and clinically meaningful patterns (**Figures 6A, B**). As highlighted in **Figure 6C** of both Case 1 and Case 2, which provide enlarged views of the other vessels segmentation, identifiable trajectories of the obturator artery (passing through the obturator canal), the internal pudendal artery (toward the perineum), and the uterine artery (toward the uterus) were observed in several test cases. The gluteal artery was consistently identifiable in all automated segmentation cases and was more clearly distinguishable than other small-diameter vessels within the other vessels category, indicating the model’s robust capacity to reconstruct this particular branch.

**Figure 7:**
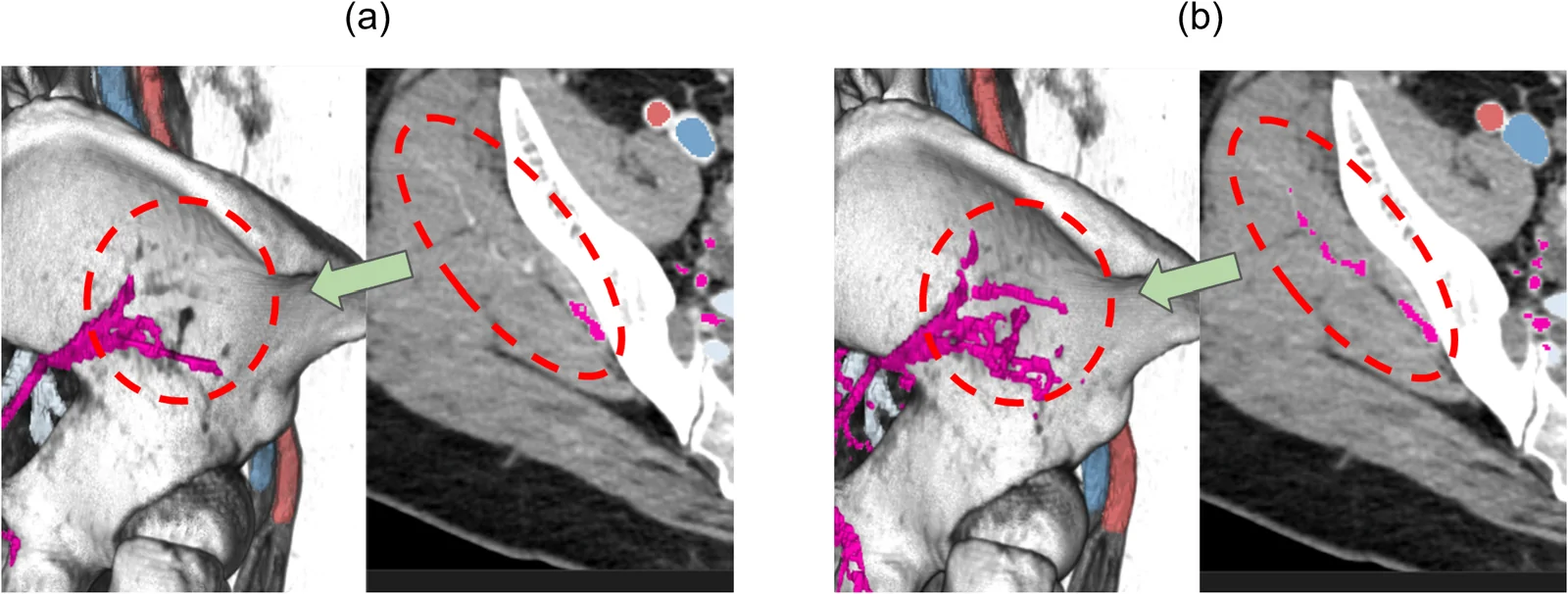
Qualitative comparison of ground truth and automated segmentation highlighting unannotated vascular structures. **(A)** 3D visualization of the ground truth, illustrating the absence of annotations in the distal region of the superior gluteal artery (SGA). **(B)** The corresponding automated segmentation mask generated by the nnU-Net v2 model.

Interestingly, qualitative evaluation revealed instances where the automated model successfully segmented vascular structures that were omitted during manual ground truth (GT) annotation. Specifically, the distal portions of the superior and inferior gluteal arteries were frequently identified by the model despite being absent from the corresponding GT masks (**Figure 7**). Retrospective review with adjusted window width and level (WW/WL) confirmed the presence of vascular structures in these regions. This finding is particularly significant, as it demonstrates the model’s robust capacity to learn anatomical context and maintain vascular continuity, effectively surpassing the manual annotations in certain complex regions.

## 5 Discussion

This study evaluated the feasibility and accuracy of automated 3D segmentation of pelvic vascular structures using nnU-Net v2 in patients with gynecologic cancer. In particular, we focused on segmenting not only major arteries and veins but also the smaller branches of the IIA and IIV, which are often clinically significant yet difficult to identify intraoperatively. While most prior studies have been limited to large vessels such as the aorta, CIA, or EIA, the automated segmentation in this work systematically incorporated multiple major branches of the IIA and IIV. This broader inclusion represents a notable advancement over existing literature (24, 26), where segmentation attempts for such small and surgically critical vessels have been extremely limited. For challenging branches such as the obturator and uterine arteries, the segmented results occasionally showed discontinuities but still allowed their anatomical pathways to be identified. The results highlight both the strengths and limitations of deep learning–based segmentation in this anatomically complex and variable region.

One particularly notable finding is that the IIV, excluding the other vessels category, showed the lowest DSC (0.585) among all target vessels. This aligns with clinical experience, as the IIV and its branches are frequently cited by gynecologic surgeons as being particularly difficult to identify during pelvic procedures. This observation suggests that the low performance on IIV may not be solely attributable to model architecture or limited training data, but rather to inherent anatomical complexity. Unlike larger or more rigid vessels, the IIV is anatomically intricate—possibly even more so than the IIA—and its walls are less rigid, making it more susceptible to deformation or obscuration by surrounding tissues. These characteristics may also have contributed to uncertainty during manual labeling. As deep learning models improve and venous segmentation becomes more reliable, structures like the IIV may eventually become key targets for enhancing the safety and precision of gynecologic cancer surgeries.

Despite the encouraging results, our study has several limitations. First, this study relied exclusively on a deep learning–based approach for vascular segmentation. Recent literature has pointed out that deep learning models alone may be insufficient to accurately reconstruct fine, branching vascular networks, especially when vessel continuity is disrupted by low contrast or imaging noise (27, 28). In our results, although branches such as the obturator and uterine arteries were identifiable, their predicted trajectories often showed discontinuities, reducing their anatomical fidelity. Preliminary investigations were conducted using a conventional centerline reconstruction method (29). However, inhomogeneous contrast agent concentration in thin vessels caused the vascular pathways to appear disconnected, significantly limiting the effectiveness of traditional intensity-based reconstruction techniques. Consequently, it was determined that robust feature-based reconstruction methods are necessary to overcome these challenges. Developing and validating such advanced learning-based post-processing frameworks constitutes a substantial, independent research endeavor, which extends beyond the scope of this initial feasibility study.

Second, the rigorous quantitative evaluation of the model is limited by the inherent incompleteness of the manual ground truth (GT) annotations, particularly within the other vessels category. Due to the complex and subtle nature of these small branches, manual annotators occasionally missed distal segments, such as parts of the superior and inferior gluteal arteries. As highlighted in the qualitative results, the automated model successfully identified many of these unannotated regions. While this demonstrates the model’s robust feature-learning capability, it paradoxically introduces uncertainty into the performance metrics. Specifically, mathematical overlap scores (e.g., DSC) penalize the model for segmenting true anatomical structures that are absent from the flawed GT masks. This suggests that the current quantitative results may underestimate the model’s true clinical utility due to the limitations of the retrospective manual labeling. Conversely, actual misclassifications also occurred within the “other vessels” category. In some instances, the model fused adjacent small arterial and venous branches into a single structure due to their tight, intertwined trajectories in the pelvic cavity. It occasionally misclassified non-target small vessels, such as the renal or ovarian arteries, as “other vessels” because of their similar small diameters and tortuous morphologies.

To address these limitations and advance toward routine clinical implementation, several future work directions are proposed. Subsequent studies must focus on developing and integrating advanced feature-based post-processing algorithms. For instance, Graph Neural Networks (GNNs) serve as a promising alternative to effectively model vascular topology, resolve discontinuities, and improve anatomical fidelity for small, tortuous branches. Furthermore, because the current model was trained and evaluated using data from a single institution, expanding the training and testing datasets to include multi-center data from diverse clinical sites is essential. Such expansion will be crucial for validating the model’s robustness and creating a highly generalizable tool suitable for broad clinical settings.

## 6 Conclusion

This study demonstrated the feasibility of automated 3D segmentation of pelvic vascular structures, including both major vessels and small-diameter branches, using the nnU-Net v2 framework. The proposed model achieved an overall average DSC of 0.756 and a MSD of 2.847 mm across nine target vascular classes. The model showed strong performance for large vessels, such as the aorta (DSC 0.952) and external iliac artery (DSC 0.867), and successfully reconstructed anatomically meaningful patterns in clinically important regions like the obturator and gluteal arteries within the other vessels category (DSC 0.467). However, the presence of fragmented morphology in certain branches highlights both the limitations of current manual labeling and the need for advanced feature-based post-processing algorithms alongside multi-center validation in future work. Ultimately, this automated segmentation framework provides a reliable foundation for enhancing preoperative surgical planning, anatomical education, and intraoperative safety in complex pelvic surgeries.

## Data Availability

The data analyzed in this study is subject to the following licenses/restrictions: The datasets used during the current study are not publicly available because of patient consent and privacy constraints. Nevertheless, upon reasonable request and subject to institutional and ethical approval, they may be made available by the corresponding author after further discussion. Requests to access these datasets should be directed to CP, chanung1319@skia.kr and KL, hohoho@catholic.ac.kr.
