# Correction: Deep learning accurately and reliably segments pelvic vascular structure in CT scans of gynecologic cancer patients

**DOI:** 10.3389/fonc.2026.1965532

**Published:** 2026-08-24

**Authors:** Hyeonseung Kim, Min Jin Jeong, Kyoyeong Koo, Youn Jin Choi, Eun Seo Heo, Woohyun Nam, Sangyun Kang, U-Young Lee, Yi-Suk Kim, Keun Ho Lee, Chan-Ung Park

**Affiliations:** 1 SKIA, Inc., Seoul, Republic of Korea; 2 Department of Obstetrics and Gynecology, Eunpyeong St. Mary’s Hospital, College of Medicine, The Catholic University of Korea, Seoul, Republic of Korea; 3 Department of Obstetrics and Gynecology, Seoul St. Mary’s Hospital, The Catholic University of Korea, Seoul, Republic of Korea; 4 The Catholic Institute for Applied Anatomy, Department of Anatomy, College of Medicine, The Catholic University of Korea, Seoul, Republic of Korea

**Keywords:** blood vessels, computed tomography angiography, deep learning, female, genital neoplasms, iliac artery

An incorrect **Funding** statement was provided. The correct funder is National Cancer Center (NCC). The correct **Funding** statement reads:

This study was supported by the National R&D Program for Cancer Control through the National Cancer Center (NCC) funded by the Ministry of Health & Welfare, Republic of Korea (No. RS-2023-CC138673).

The original version of this article has been updated.

